# Serum sodium levels associate with recovery of kidney function in immune checkpoint inhibitor nephrotoxicity

**DOI:** 10.3389/fmed.2023.1020691

**Published:** 2023-07-20

**Authors:** Désirée Tampe, Eva Baier, Samy Hakroush, Björn Tampe

**Affiliations:** ^1^Department of Nephrology and Rheumatology, University Medical Center Göttingen, Göttingen, Germany; ^2^Institute of Pathology, University Medical Center Göttingen, Göttingen, Germany; ^3^SYNLAB Pathology Hannover, SYNLAB Holding Germany, Augsburg, Germany

**Keywords:** PD-L1, PD-1, checkpoint inhibition, kidney injury, kidney function recovery, serum sodium

## Abstract

**Background:**

Immune checkpoint inhibitors (ICIs) are novel drugs targeting programmed cell death protein 1-ligand 1 (PD-L1) or its receptor (PD-1). Enhancing the immune system has also been associated with a wide range of immune-related adverse events (irAE). Among them, acute interstitial nephritis (AIN) is a rare but deleterious irAE in the kidney. However, determinants of recovery and long-term kidney function after ICI withdrawal and steroid therapy thereafter remain elusive. Therefore, we here aimed to identify parameters associated with recovery of kidney function in this previous established cohort of AIN in the context of ICI therapy.

**Methods:**

We here monitored kidney function over a mean follow-up time of 812 days in comparison with clinical, histopathological and laboratory parameters associated with recovery of kidney function after AIN related to ICI nephrotoxicity.

**Results:**

Abundance of intrarenal PD-L1/PD-1 did not correlate with recovery of kidney function. Furthermore, cumulative steroid dose that was initiated for treatment of AIN related to ICI nephrotoxicity was also not associated with improvement of kidney function. Finally, chronic lesions in the kidney including glomerular sclerosis and interstitial fibrosis/tubular atrophy (IF/TA) did not correlate with eGFR change during the follow-up time. However, we here identified that lower levels of serum sodium at time of kidney biopsy were the strongest independent predictor of renal recovery in ICI-related nephrotoxicity.

**Conclusion:**

Because low serum sodium levels associated with better improvement of kidney function, these observations might contribute to novel approaches to enhance recovery after AIN related to ICI nephrotoxicity.

## Introduction

Immune checkpoint inhibitors (ICIs) are novel drugs targeting programmed cell death protein 1-ligand 1 (PD-L1, synonym CD274, B7 homolog 1) or its receptor (PD-1, synonym CD 279), and are increasingly used in cancer therapy (1). Allowing T cells to remain an activated state by blocking negative co-stimulatory pathways, ICIs enhance the anti-tumoral immune response. However, enhancing the immune system has also been associated with a wide range of immune-related adverse events (irAE) (2). Among them, acute interstitial nephritis (AIN) is a rare but severe irAE in the kidney and the most common biopsy-proven diagnosis in ICI-related nephrotoxicity (3, 4). We have recently characterized abundance of PD-L1 and its receptor PD-1 in a cohort of biopsy-proven AIN related to nephrotoxicity of ICI therapy presenting with acute kidney injury (AKI) (5). While glomerular PD-L1 correlated with kidney function and interstitial cell PD-1 positivity specifically with severity of kidney injury, determinants of long-term kidney function after ICI withdrawal and steroid therapy thereafter remain elusive (5). Therefore, we here aimed to identify parameters associated with recovery of kidney function in this previous established cohort of AIN in the context of ICI therapy by monitoring of long-term kidney function (5).

## Methods

### Study population

A total number of 6 cases with biopsy-proven AIN related to nephrotoxicity of ICI therapy with a long-term follow-up were retrospectively included between 2015 to 2020 at the University Medical Center Göttingen, Germany (Table 1). The patient cohort has previously been described (5, 6). The studies involving human participants were reviewed and approved by the Institutional Review Board of the University Medical Center Göttingen, Germany (no. 22/2/14). The patients/participants provided their written informed consent for the use of routinely collected data for research purposes as part of their regular medical care in the contract of the University Medical Center Göttingen. Medical records were used to obtain data on age, sex, medication, date of biopsy, laboratory results and urinary analysis. At the time of kidney biopsy, 3/6 (50%) of patients received a proton pump inhibitor (PPI), and none a non-steroidal anti-inflammatory drug (NSAR) or antibiotic (Table 1). For assessment of kidney function recovery, difference between worst estimated glomerular filtration rate (eGFR) at time of kidney biopsy and last documented eGFR during follow-up was calculated. The mean follow-up time was 812 days, and 5/6 (83.3%) of patients showed improvement of kidney function (Table 1).

**Table 1:** Clinical, laboratory, and histopathological parameters of the total cohort.

Clinical data	Value
Female sex – no. (%)	2 (33.3)
Age – years	72.2 ± 7.7
PD-1 targeted therapy – no. (%)	5 (83.3)
PD-L1 targeted therapy – no. (%)	1 (16.7)
Pembrolizumab – no. (%)	2 (33.3)
Nivolumab – no. (%)	3 (50)
Durvalumab – no. (%)	1 (16.7)

Laboratory parameters	Value	Normal range
Hemoglobin – g/dL	10.8 ± 1.8	13.5–17.5
Platelets – x10^3^/μL	265.8 ± 58.8	150–350
Leukocytes – x10^3^/μL	8.4 ± 2.6	4–11
TSH – mIU/L	0.9 ± 0.8	0.35–4.94
Sodium – mmol/L	138.7 ± 0.8	136–145
Chloride – mmol/L	106.8 ± 1.7	97–108
Potassium – mmol/L	4.4 ± 0.5	3.5–4.6
Calcium – mmol/L	2.2 ± 0.2	2.2–2.55
Serum creatinine – mg/dL	3.5 ± 1.8	0.7–1.2
eGFR – mL/min	21.3 ± 12.2	> 60
BUN – mg/dL	50.2 ± 21.6	8–26
CRP – mg/L	38.1 ± 38.4	≤ 5
LDH – U/L	317.3 ± 58.6	125–250
Complement C3c – g/L	1.2 ± 0.2	0.82–1.93
Complement C4 – g/L	0.3 ± 0.1	0.15–0.57

Urinary parameters	Value	Normal range
Proteinuria – mg/L	206.5 ± 179	< 140
Albuminuria/g creatinine – mg/g	100.6 ± 91.3	< 30
Urinary α_1_-microglobulin – mg/L	41.3 ± 26.7	< 12
Urinary IgG – mg/L	12.2 ± 8.8	<9.6
Urinary kappa – mg/L	32 ± 20	< 6.8
Urinary lambda – mg/L	9.2 ± 5.1	< 3.7
Urinary kappa/lambda – ratio	3.8 ± 1.9	> 1 or < 5.2

Histopathological findings	Value
Glomerular sclerosis – % of total	12 ± 11.6
IF/TA – %	40 ± 20
C1q positivity – no. (%)	0 (0)
C3c positivity – no. (%)	0 (0)
Co-medication	
PPI – no. (%)	3 (50)
NSAR – no. (%)	0 (0)
Antibiotic – no. (%)	0 (0)
Treatment regimen	
ICI withdrawal – no. (%)	6 (100)
Steroids – no. (%)	6 (100)
Cumulative steroid dose – mg	3,369 ± 1902
Follow-up data	
Follow-up time – days	812 ± 352
Last eGFR – mL/min	41.2 ± 16.1
eGFR recovery – mL/min	22.9 ± 16

Continuous and ordinal variables were presented as mean ± standard deviation, categorical variables as percentages of total. AIN, acute interstitial nephritis; BUN, blood urea nitrogen; C1q, complement factor 1q; C3c, complement factor 3 conversion product; C4, complement factor 4; CRP, C-reactive protein; eGFR, estimated glomerular filtration rate; IF/TA, interstitial fibrosis/tubular atrophy; IgG, immunoglobulin G; LDH, lactate dehydrogenase; no., number; NSAR, non-steroidal anti-inflammatory drug; PD-L1, programmed cell death protein 1-ligand 1; PPI, proton pump inhibitor; TSH, thyroid stimulating hormone.

### Renal histopathology and immunohistochemistry

A renal pathologist evaluated the kidney biopsies and was blinded to clinical data. After deparaffinization in xylene and rehydration in ethanol containing distilled water, formalin-fixed, paraffin-embedded kidney sections were stained using primary antibodies against PD-L1 (1:100, ab205921, Abcam, Cambridge, UK), PD-1 (1:500, ab52587, Abcam, Cambridge, UK), C1q (1:30,000, A0136, Agilent Dako, Santa Clara, USA), and C3c (1:10,000, A0062, Agilent Dako, Santa Clara, USA), labeling was performed using Novolink™ Polymer Detection System (Leica Biosystems, Wetzlar, Germany) according to the manufacturer’s protocol. Nuclear counterstain was performed by using Mayer’s Hematoxylin Solution (Sigma, St. Louis, USA). As previously described, the intensity of PD-L1 staining was evaluated at 400x magnification and scored semiquantitatively (0: no staining, 1: weak and segmental staining, 2: moderate staining, 3: strong staining). Interstitial cells positive for PD-1 were evaluated by using mean values of 10 randomly selected cortical visual fields at 400x magnification and scored semiquantitative (0, no cell per visual field, 1: 1–3 cells per visual field, 2: 3–6 cells per visual field, 3: >6 cells per visual field) (5).

### Plasma C3c and C4 measurements

Plasma concentrations of human complement components C3c (9D9621, Abbott, Chicago, USA) and C4 (9D9721, Abbott, Chicago, USA) were determined by turbidimetric measurements on the ARCHITECT-C module.

### Statistical methods

Variables were tested for normal distribution using the Shapiro–Wilk test. Statistical comparisons were not formally powered or prespecified. Continuous and ordinal variables were presented as mean ± standard deviation, categorical variables as percentages of total. Spearman’s correlation was performed to assess correlations and heatmaps reflect the mean values of Spearman’s ρ. A Spearman’s ρ more than ±0.9 in the correlation matrix was defined as relevant indicated by rectangle boxes, and independent statistical evaluation of these parameters was performed by multiple linear regression. A probability (*p*) value of <0.05 was considered statistically significant. Data analyses were performed with GraphPad Prism (version 8.4.3 for MacOS, GraphPad Software, San Diego, California, USA), regression analyses were performed using IBM SPSS Statistics (version 27 for MacOS, IBM Corporation, Armonk, NY, USA).

## Results

**Figure 1:**
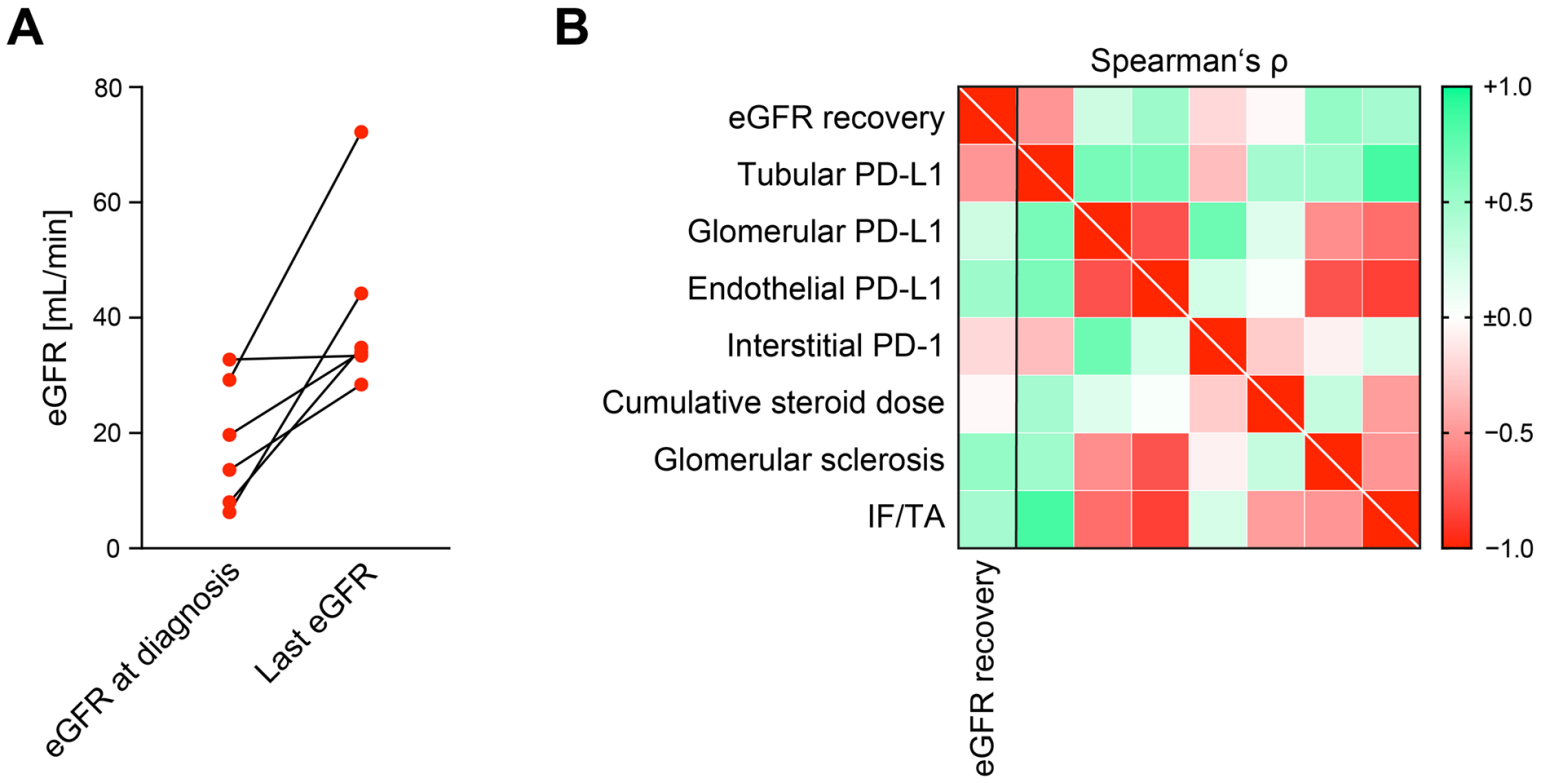
Histopathological lesions or cumulative steroid dose do not affect renal recovery after AIN related to ICI nephrotoxicity. **(A)** Difference between worst eGFR at time of diagnosis and last documented eGFR during follow-up in the patient cohort are shown. **(B)** Intensity of PD-L1/PD-1 positivity within different renal compartments, cumulative steroid dose and histopathological findings in association with eGFR recovery in AIN related to ICI nephrotoxicity are shown by heatmap reflecting mean values of Spearman’s ρ. AIN, acute interstitial nephritis; eGFR, estimated glomerular filtration rate (CKD-EPI); ICI, immune checkpoint inhibitor; IF/TA, interstitial fibrosis/tubular atrophy; PD-1, programmed cell death protein 1; PD-L1, programmed cell death protein 1-ligand 1.

**Figure 2:**
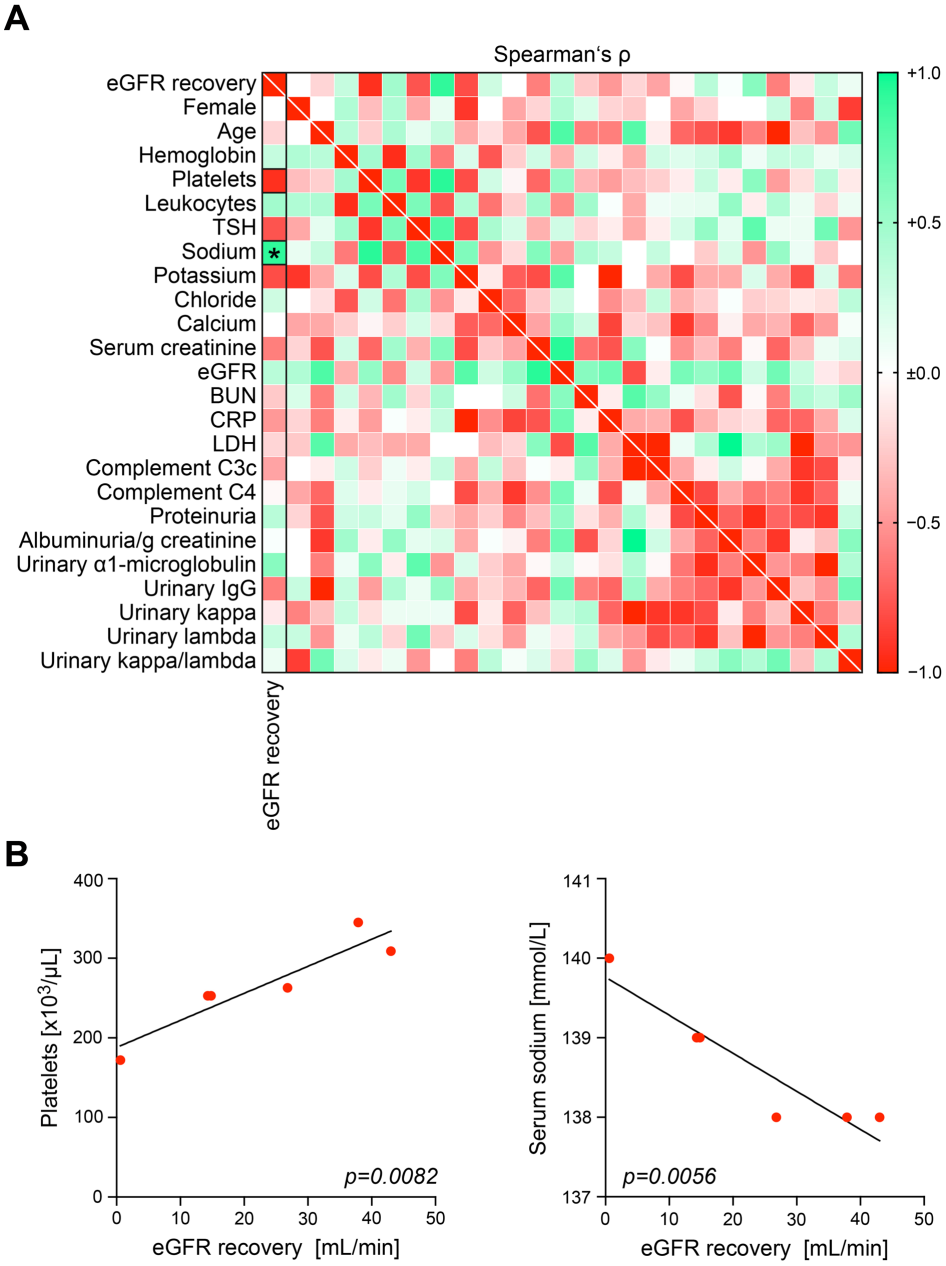
Lower serum sodium levels correlate with recovery of kidney function after AIN related to ICI nephrotoxicity. **(A)** Recovery of eGFR during follow-up in association with clinical and laboratory parameters are shown by heatmap reflecting mean values of Spearman’s ρ. Rectangle boxes indicate a Spearman’s ρ more than ±0.9, the asterisk a significant correlation in the stepwise linear regression analysis (*p < 0.05*). **(B)** Simple linear regression analysis for platelet counts and serum sodium levels in association with eGFR recovery in the patient cohort. AIN, acute interstitial nephritis; BUN, blood urea nitrogen; C3c, complement factor 3 conversion product; C4, complement factor 4; CRP, C-reactive protein; eGFR, estimated glomerular filtration rate; ICI, immune checkpoint inhibitor; IgG, immunoglobulin G; LDH, lactate dehydrogenase; TSH, thyroid stimulating hormone.

We included a total number of 6 kidney specimens with ICI-related nephrotoxicity (5 cases with ICI therapy targeting PD-1 and 1 targeting PD-L1, Table 1). In all cases, ICI treatment was discontinued, and steroids were initiated (Table 1). The mean follow-up time was 812 days, and 5/6 (83.3%) of patients showed improvement of kidney function (Figure 1A). Based on our previous observations that intrarenal PD-L1 and PD-1 positivity was associated with kidney injury, we first analyzed its association with renal recovery in this cohort (5). Abundance of intrarenal PD-L1/PD-1 did not correlate with recovery of kidney function (Figure 1B). Furthermore, cumulative steroid dose that was initiated for treatment of AIN related to ICI nephrotoxicity was also not associated with improvement of kidney function (Figure 1B). Finally, chronic lesions in the kidney including glomerular sclerosis and interstitial fibrosis/tubular atrophy (IF/TA) did not correlate with eGFR change during the follow-up time (Figure 1B). We next analyzed recovery of kidney function in association with clinical and laboratory parameters at time of kidney biopsy. Platelet counts were positively associated with eGFR recovery (Spearman’s ρ = 0.9276), and sodium levels negatively with recovery of kidney function (Spearman’s ρ = −0.9258, Figure 2A). These observations were confirmed by simple linear regression analysis for platelet counts (*p* = 0.0082) and serum sodium levels (*p* = 0.0056, Figure 2B). As confirmed by multiple regression analysis comparing the association between eGFR recovery and identified parameters, lower levels of serum sodium at time of kidney biopsy were the strongest independent predictor of renal recovery in ICI-related nephrotoxicity (Table 2). In summary, lower serum sodium levels correlated with recovery of kidney function after AIN related to ICI nephrotoxicity independent of histopathological lesions or cumulative steroid dose.

**Table 2:** Multiple linear regression analyses correlating with eGFR recovery.

Comparison with eGFR recovery	ß	*p* value
Sodium – mmol/L	−0.9381	0.0056
Thrombocytes – x10^3^/μL	0.4339	0.3123

eGFR, estimated glomerular filtration rate (CKD-EPI); PD-1, programmed cell death protein 1.

## Discussion

We have recently characterized abundance of PD-L1 and its receptor PD-1 in a cohort of biopsy-proven AIN related to nephrotoxicity of ICI therapy presenting with acute kidney injury (AKI) (5). While glomerular PD-L1 correlated with kidney function and interstitial cell PD-1 positivity specifically with severity of kidney injury, determinants of long-term kidney function after ICI withdrawal and steroid therapy thereafter remain elusive (5). Therefore, we here monitored kidney function over a mean follow-up time of 812 days and identified higher platelet counts and low serum sodium levels to be associated with improved renal recovery after AIN related to ICI nephrotoxicity. An inverse correlation between platelet counts and serum sodium levels has already been described, and we here confirm that serum sodium at time of kidney biopsy was the strongest independent predictor of renal recovery in ICI-related nephrotoxicity (7). Higher dietary salt consumption in populations with chronic kidney disease (CKD) has already been associated with proteinuria as independent risk factors for CKD progression (8). While it has been proposed that small increases in serum sodium levels are of importance, the mechanisms underlying these adverse effects of sodium are incompletely understood (9, 10). Besides effects attributed to altered blood pressure affected by serum sodium, there is *in vitro* evidence that small increases in sodium concentrations directly impact endothelial cell homeostasis by damage to the glycocalyx layer and inhibition of nitric oxide release (11, 12). Furthermore, increased sodium concentrations have also been shown to enhance transcription induction of pro-fibrotic mediators including transforming growth factor-beta 1 (TGF-β1), associated with progressive CKD (13). Interestingly, it has recently been shown that a 5 mmol/L increase in baseline serum sodium was associated with an eGFR loss of 7.4 mL/min over 6 years in a population with established CKD (14). These findings support that serum sodium levels within the normal range are a risk factor for CKD progression. Consistent to that, we here identified low levels of serum sodium within the normal range associated with renal recovery after AIN related to ICI nephrotoxicity. These observations require further confirmation in independent cohorts, and restriction of salt consumption to lower serum sodium levels after AIN related to ICI nephrotoxicity would be an attractive therapeutical intervention as it has also been shown in multiple trials including CKD patients (15–21).

This study has several limitations due to the small patient number, its retrospective design, and no independent validation. Moreover, urinary sodium levels were not measured but would be of interest regarding urinary sodium excretion. However, nephrotoxicity related to ICI therapy is a rare event with only a limited number of kidney biopsies (22–24). Our observations that low serum sodium levels to be associated with better improvement of kidney function might contribute to novel approaches to enhance recovery after AIN related to ICI nephrotoxicity.

## Data availability statement

The original contributions presented in the study are included in the article/supplementary material, further inquiries can be directed to the corresponding author.

## Ethics statement

The studies involving human participants were reviewed and approved by the Ethics Committee of the University Medical Center Göttingen. The patients/participants provided their written informed consent to participate in this study.

## Author contributions

BT conceived the study, collected and analyzed data, and wrote the first draft. DT and EB collected and analyzed data. SH evaluated all kidney biopsies. All authors contributed to the article and approved the submitted version.

## Funding

BT was supported by the Research program, University Medical Center Göttingen (1403720). EB was funded by the Else-Kröner research program entitled “*molecular therapy and prediction of gastrointestinal malignancies*” (7-67-1840876). The authors also acknowledge support by the Open Access Publication Funds of the Göttingen University. The funding sources were not involved in the design, collection, analysis, interpretation, writing or decision to submit the article.

## Conflict of interest

Author SH was employed by company SYNLAB Holding Germany.

The remaining authors declare that the research was conducted in the absence of any commercial or financial relationships that could be construed as a potential conflict of interest.

## Publisher’s note

All claims expressed in this article are solely those of the authors and do not necessarily represent those of their affiliated organizations, or those of the publisher, the editors and the reviewers. Any product that may be evaluated in this article, or claim that may be made by its manufacturer, is not guaranteed or endorsed by the publisher.
